# Low Power Emission Pulse Generation Circuit Based on n-Type Amorphous In-Ga-Zn-Oxide Transistors for Active-Matrix Organic Light-Emitting Diode Displays

**DOI:** 10.3390/mi15111330

**Published:** 2024-10-30

**Authors:** Min-Kyu Chang, Ji Hoon Kim, Hyoungsik Nam

**Affiliations:** Department of Information Display, Kyung Hee University, Seoul 02447, Republic of Korea; mkchang@khu.ac.kr (M.-K.C.); gns4807@khu.ac.kr (J.H.K.)

**Keywords:** low power, emission pulse, inverter, a-IGZO TFT, depletion-mode, shoot-through current path

## Abstract

This paper presents a low power emission (EM) pulse generation circuit using n-type amorphous In-Ga-Zn-Oxide (a-IGZO) semiconductor thin-film transistors (TFTs). The low power consumption is achieved by avoiding the shoot-through current paths through an optimized inverter circuit. The proposed circuit consists of 12 TFTs and 2 capacitors including 6 TFTs and 1 capacitor for the inverter circuit to control the pulling-down TFTs. In addition, the wider variance range of the threshold voltage ($V_{\mathrm{th}}$) from −4 V to 2.5 V is covered by additional 6 TFTs for series-connected two transistor (STT) schemes and two low supply voltages to take into account the negative $V_{\mathrm{th}}$ of depletion-mode TFTs. The simulation of 30 EM circuits is conducted over a 6.1-inch active-matrix organic light-emitting diode display of 120 Hz refresh rate and 3840 × 2160 (UHD) resolution. The power consumption of the EM circuit with the proposed inverter is measured at the low values from 0.836 mW to 0.568 mW over pulse widths from 3 to 2157 horizontal times. It is ensured that the proposed circuit achieves the low power consumption regardless of pulse widths.

## 1. Introduction

Flat panel displays have played a crucial role in the transmission and representation of visual information. Particularly, in consumer electronics such as mobile devices and televisions, the performance metrics of displays such as resolution, frame rate, and peak brightness are key determinants for enhancing user experience [1,2]. In recent years, active-matrix organic light-emitting diode (AMOLED) displays have been widely adopted due to their superior image qualities and small form factor [3,4,5,6]. Since the battery usage time without recharging is critical for mobile devices, power-efficient technologies have become more essential [7,8,9]. Among power-efficient thin-film transistor (TFT) backplane technologies, amorphous In-Ga-Zn-Oxide (a-IGZO) TFTs are characterized by low leakage currents which can minimize power consumption [10,11,12,13,14,15]. Whereas low-temperature polycrystalline silicon (LTPS) TFTs have been widely used in mobile displays due to their high mobility and reliability, high leakage current makes the low frequency driving impossible for low power consumption [16,17,18], leading to the increased adoption of a-IGZO TFTs in mobile devices and other electronic devices.

In addition to the low power, the low cost implementation has been one of key driving forces in the display industries by integrating circuits in TFT-based panels. This integration enables the cost reduction by excluding the external integrated circuits [19,20,21]. Therefore, the low power technologies for the integrated TFT circuits have been also developed along with the stability improvement [22,23,24,25]. Among those integrated circuits, the emission pulse (EM) generation circuit is an additional driving circuit to the gate driver of the scanning pulse generation in order to manage the emission periods of OLEDs for pixel compensation and current adjustment [26,27,28,29,30]. The EM drivers also generate the shifted pulses like the usual shift registers, but their pulse widths are much longer than a horizontal time of a normal scanning pulse. Therefore, the EM drivers should contain inverters [31,32] to keep the pulling-down TFTs turned off stably during the high pulse generation, where the inverters composed of one-type TFTs may increase power consumption proportionally to the pulse width [33]. To get rid of this dependency between power consumption and pulse width, it is essential to avoid the occurrence of the shoot-through current paths. In this paper, we propose an EM pulse generation circuit that employs the internal inverter without any occurrences of shoot-through current paths. As a result, the low power consumption is maintained without any increases over the pulse width variation.

## 2. Proposed Low Power EM Pulse Generation Circuit

**Figure 1:**
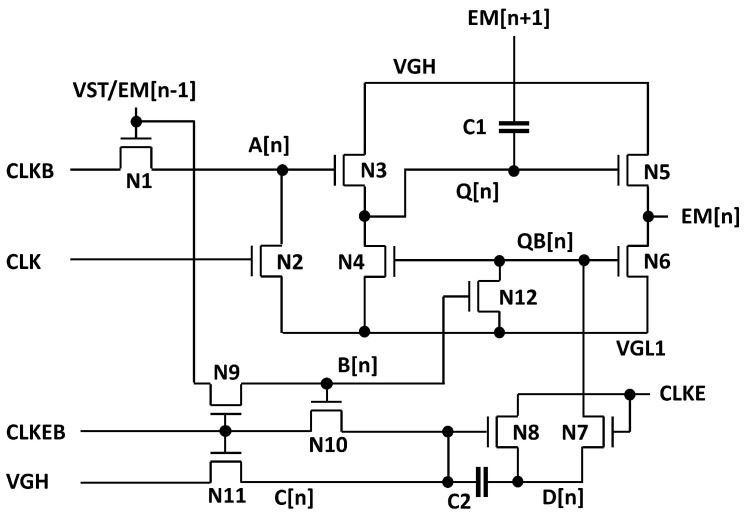
The basic structure of the proposed EM pulse generation circuit (12T2C).

**Figure 2:**
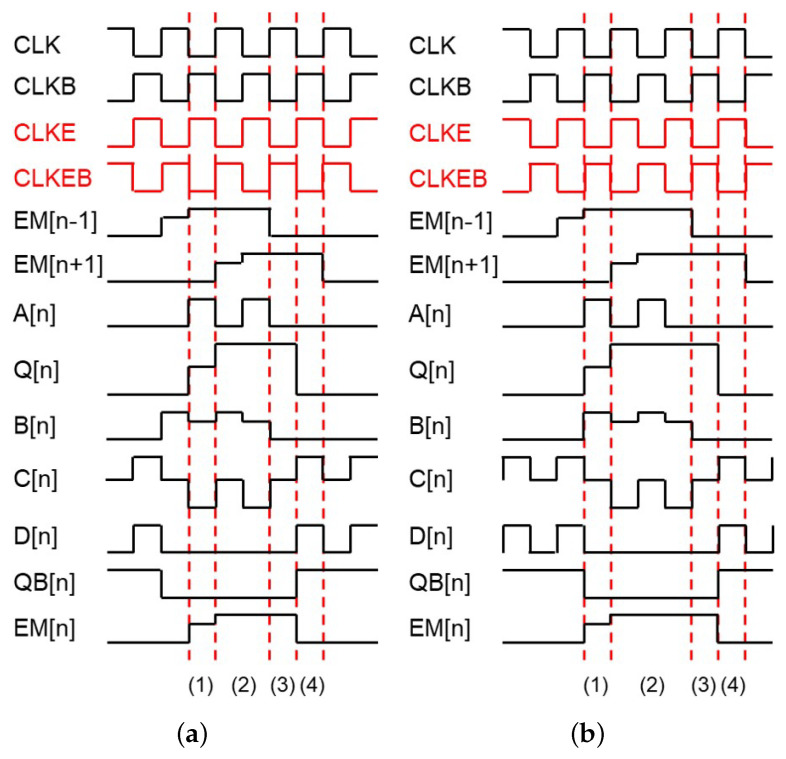
Timing diagrams for the proposed EM pulse generation circuit. (**a**) Even multiple pulse width of a horizontal time (4 horizontal times) where CLKE is equivalent to CLKB. (**b**) Odd multiple pulse width of a horizontal time (5 horizontal times) where CLKE is synchronized to CLK. The operation consists of (1) Pulling-Up, (2) Capacitive-Coupling, (3) Holding, and (4) Pulling-Down periods.

The basic circuit schematic of the proposed EM pulse generation circuit is illustrated in Figure 1 which consists of 12 n-type TFTs (N1 to N12) and 2 capacitors (C1, C2), where all TFTs are assumed as enhancement-mode. Especially, 6 TFTs from N7 to N12 and C2 are employed for the inverter to adjust the voltage of QB[n] without any shoot-through current paths. The basic operating principle is based on the previous EM circuit [33] to support any multiple EM pulse widths of a horizontal time as illustrated in Figure 2 for two cases of even and odd multiples. CLK and CLKB represent two out-of-phase main clocks with the pulse width of a horizontal time. VGH and VGL1 denote high and low supply voltages, respectively. CLKE and CLKEB are used as additional out-of-phase clock signals to support various EM pulse widths of even and odd multiples over a horizontal time. While the odd multiple widths are realized by CLKE and CLKEB in phase with CLK and CLKB, even multiple widths are generated by CLKE and CLKEB synchronized to CLKB and CLK, respectively. EM[n] represents the output of the EM driver at the n-th line, while EM[n − 1] and EM[n + 1] represent the outputs of its previous and next stages.

N1 and N2 are used to charge and discharge A[n] to control N3. While N3 and N5 are responsible for pulling up Q[n] and EM[n], N4 and N6 pull down Q[n] and EM[n]. N12 handles charging and discharging at QB[n] and N7 and N8 adjust the timing to turn off Q[n] and EM[n] one horizontal time later by CLKE and CLKEB. C1 is a coupling capacitor to pull Q[n] up during the rising transition of EM[n + 1] for the stable output at VGH. C2 serves as a storage capacitor for C[n] and D[n].

The main operation consists of four steps such as (1) Pulling-Up, (2) Capacitive-Coupling, (3) Holding and (4) Pulling-Down. The behavior of each step for the even multiple pulse width of a horizontal time is described including voltages of important nodes.

**Figure 3:**
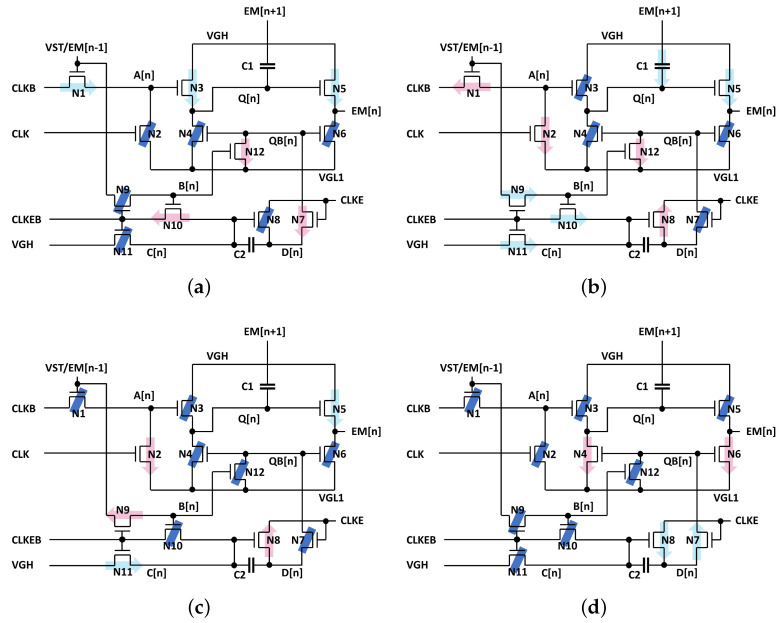
The four-phase operational sequence of the proposed EM circuit. (**a**) Pulling-Up, (**b**) Capacitive-Coupling, (**c**) Holding, (**d**) Pulling-Down. Blue line, light blue arrow, and pink arrow represent turned-off TFT, charging, and discharging, respectively.

(1) Pulling-Up: EM[n − 1], CLK, CLKB, CLKE, and CLKEB are asserted at high, low, high, high, and low voltage levels as shown in Figure 3a. CLKB is set to high one horizontal time later than EM[n − 1], charging A[n] to $\mathrm{VGH}-V_{th1}$ through N1. Q[n] is charged to $\mathrm{VGH}-V_{th1}-V_{th3}$ by N3, pulling up EM[n] to $\mathrm{VGH}-V_{th1}-V_{th3}-V_{th5}$ through N5. Consequently, the rising transition of EM[n] is delayed by one horizontal time, compared to EM[n − 1].
  One horizontal time before this step, B[n] has been charged to high by the high EM[n −1] pulse. Therefore, during the *Pulling-Up* step, since N8, N9, and N11 are turned off, D[n] and QB[n] are discharged into VGL1 through N7 and N12, respectively, turning two pulling-down TFTs of N4 and N6 off.
(2) Capacitive-Coupling: EM[n − 1], CLK, CLKB, CLKE, and CLKEB turn to be high, high, low, low, and high as depicted in Figure 3b. A[n] is discharged into VGL1 through N2 and N1 at the high CLK and is charged via N1 by CLKB, keeping Q[n] at the high voltage level. Especially, when N3 is turned off by the low A[n], Q[n] stays at a floating node state and is boosted to $\mathrm{VGH}-V_{th1}-V_{th3}-V_{th5}+\left(\mathrm{VGH}-\mathrm{VGL}1\right)\times C_{1}/\left(C_{1}+C_{Q}\right)$ by the capacitive coupling of C1 over the rising transition of EM[n + 1]. $C_{1}$ and $C_{Q}$ are the capacitance of C1 and the parasitic capacitance seen at Q[n], respectively. Since Q[n] is boosted to sufficiently higher than VGH, N5 allows EM[n] to reach to VGH. B[n] and C[n] are charged to high through N9, N10, and N11 from EM[n − 1], CLKEB, and VGH while D[n] is retained at VGL1 through N8 by the low CLKE.
(3) Holding: Since EM[n − 1] and CLKEB are low and high, B[n] is pulled down through N9 and C[n] is kept at VGH through N11 as described in Figure 3c. N7 and N12 are turned off, holding QB[n] at the floating state of VGL1. As the low QB[n] turns N4 and N6 off, Q[n] and EM[n] are allowed to stay at the high voltage.
(4) Pulling-Down: Since A[n] and B[n] are maintained at VGL1 by N2 and N9 as presented in Figure 3d, N3 and N12 are turned off. C[n] is maintained at VGH due to N10 and N11 turned off by the low voltage B[n] and CLKEB. Consequently, the high CLKE pulls QB[n] up through N7 and N8, pulling Q[n] and EM[n] down to VGL1 via N4 and N6, respectively.

**Figure 4:**
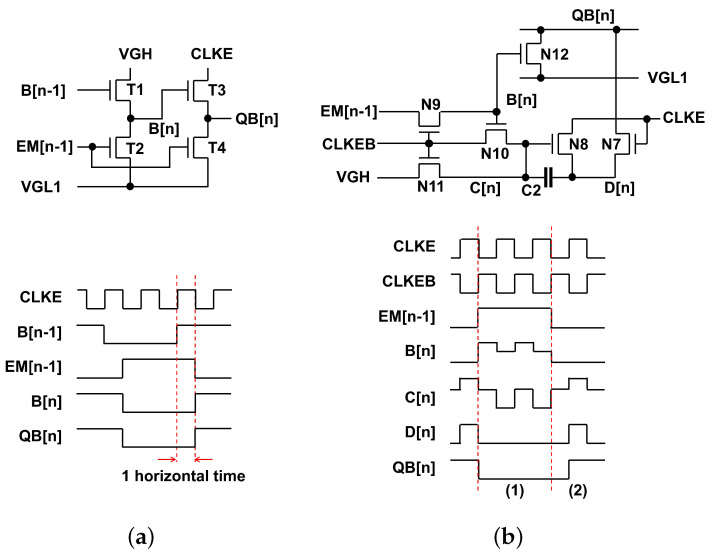
Internal inverter circuits for QB[n] generation. (**a**) Previous circuit with the shoot-through current path of one horizontal time regardless of the pulse width [33], (**b**) Proposed circuit without any shoot-through current paths.

In particular, the proposed internal inverter for QB[n] generation is compared in the previous one [33]. To avoid increasing the power consumption proportionally to the pulse width, the previous approach applies B[n − 1] that is the inverted EM[n-2] to the load TFT (T1) ensuring that the shoot-through current path occurs only for one horizontal time regardless of the pulse width as depicted in Figure 4a. However, the proposed inverter generates QB[n] without the shoot-through current path as presented in Figure 4b. In the (1) period, N12 offers the discharging path by the high B[n] that is produced by sampling EM[n − 1] one horizontal time later at the high CLKEB. Because C[n] is driven by CLKEB, D[n] and QB[n] are pulled down at VGL1 without any racing issues regarding N12. In the (2) period, QB[n] should be pulled up at one horizontal time later than the falling transition of EM[n − 1] to generate EM[n] shifted by one horizontal time. Because C[n] is kept at the high voltage, N8 is turned on and QB[n] is charged by the high CLKE through N7 and N8. Consequently, QB[n] is successfully adjusted without any occurrence of shoot-through current paths.

**Figure 5:**
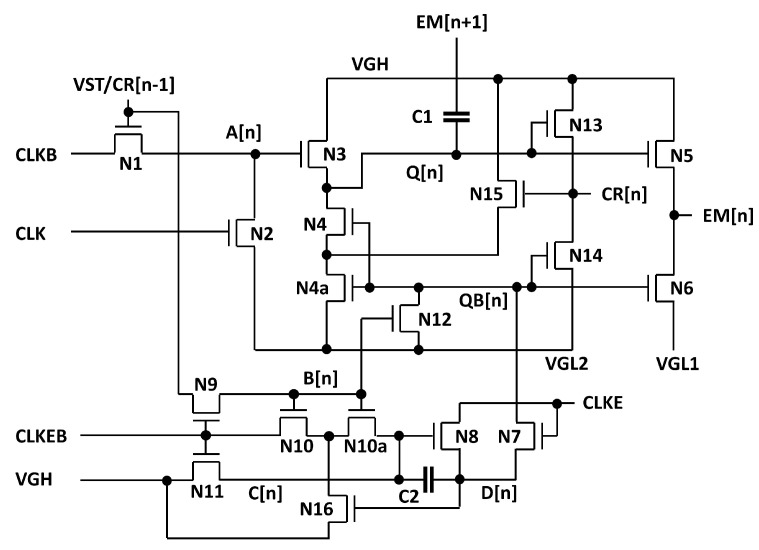
EM pulse generation circuit with depletion mode prevention structures.

In addition, the depletion-mode operation of a-IGZO TFTs is taken into account. The series-connected two transistor (STT) methods [34] are applied to N4 and N10 of Figure 1 with additional TFTs of N4a, N15, N10a, and N16 to remove the leakage current paths from Q[n] and C[n] as shown in Figure 5. Two low supply voltages of VGL1 and VGL2, where VGL2 is lower than VGL1, are also utilized to reduce the power consumption at the large sized pulling-up and pulling-down TFTs of N5 and N6. Shift operations are conducted by the carry signal (CR[n]) with the range of VGL2 to VGH.

## 3. Simulation Results

**Figure 6:**
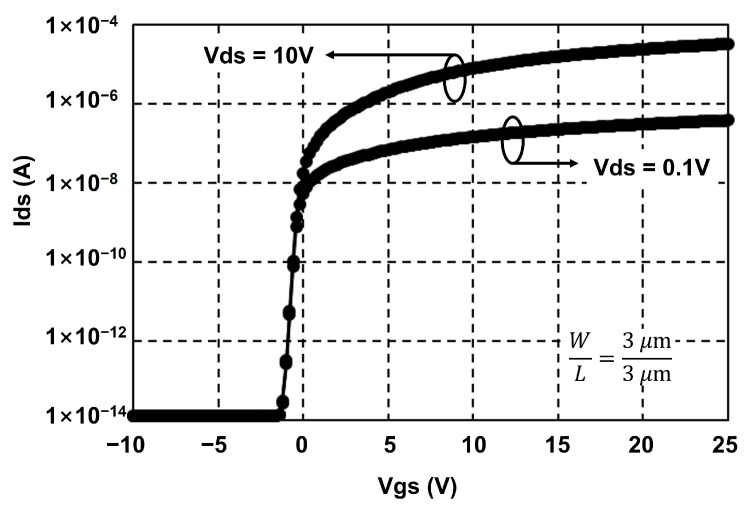
Transfer curve of a n-Type a-IGZO TFT model for simulation.

**Figure 7:**
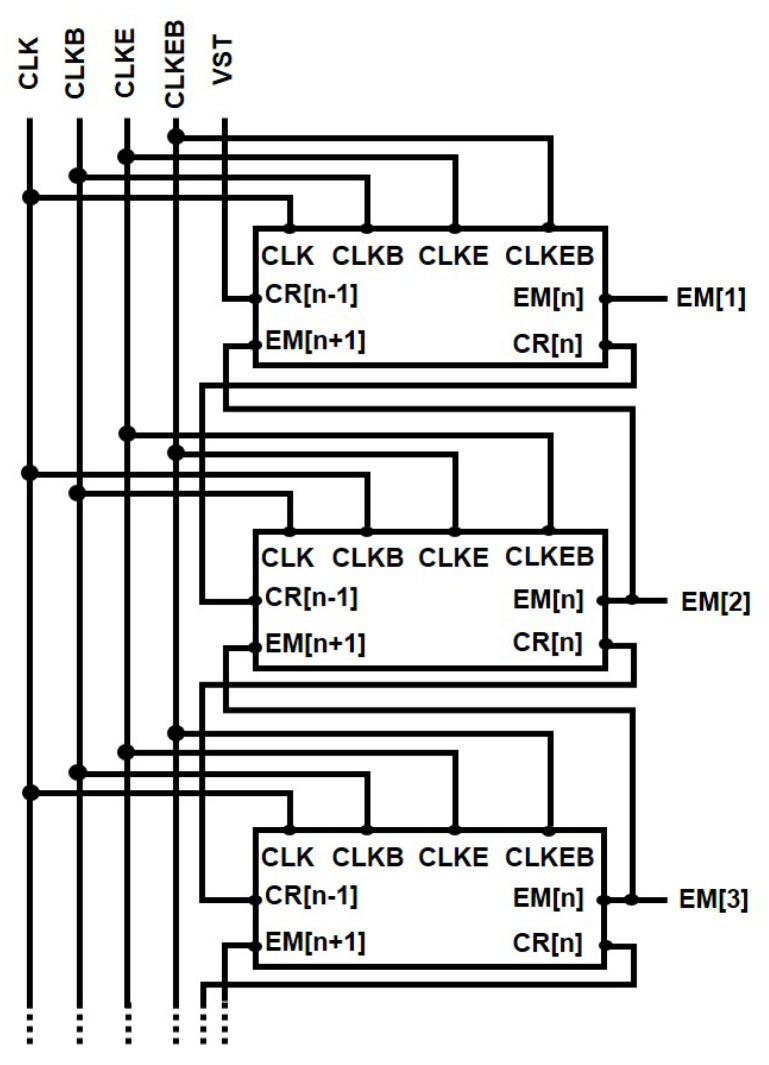
Overall configuration of proposed EM pulse generation circuits.

The proposed low power EM circuit to cope with depletion-mode operation is evaluated using a simulation program with integrated circuit emphasis (SPICE) based on a n-type a-IGZO TFT backplane that has the transfer curve shown in Figure 6. As for electrical characteristics, threshold voltage ($V_{th}$) and mobility are −0.65 V and 5.762 ${\mathrm{cm}}^{2}$/Vs, respectively. The target display is assumed as the frame rate of 120 Hz, ultra high definition (UHD) resolution of 3840×2160, and the vertical blank of 500 μs that is equivalent to one horizontal time of 3.63 μs and the clock frequency of 138 kHz. The capacitive and resistive loads at the outputs of EM circuits are set to 25 pF and 2 kΩ for a 6.1-inch display. VGH, VGL1, and VGL2 are 15 V, 0 V, and −6 V. All channel lengths of TFTs are 3 μm and channel widths of most TFTs excluding N3, N5, and N6 are also 3 μm for the small area implementation. The width of N3 is 9 μm to support sufficient charging at Q[n] and widths of N5 and N6 are 150 μm and 120 μm to drive heavy capacitive and resistive loads at EM[n]. The channel widths are summarized in Table 1. The capacitance of C1 ($C_{1}$) used for capacitive coupling of Q[n] is 50 fF, and the capacitance of C2 ($C_{2}$) employed to hold C[n] and D[n] is 10 fF. The simulation has been conducted over 30 stages of EM circuits that are configured as shown in Figure 7, where VST is a vertical start pulse.

**Figure 8:**
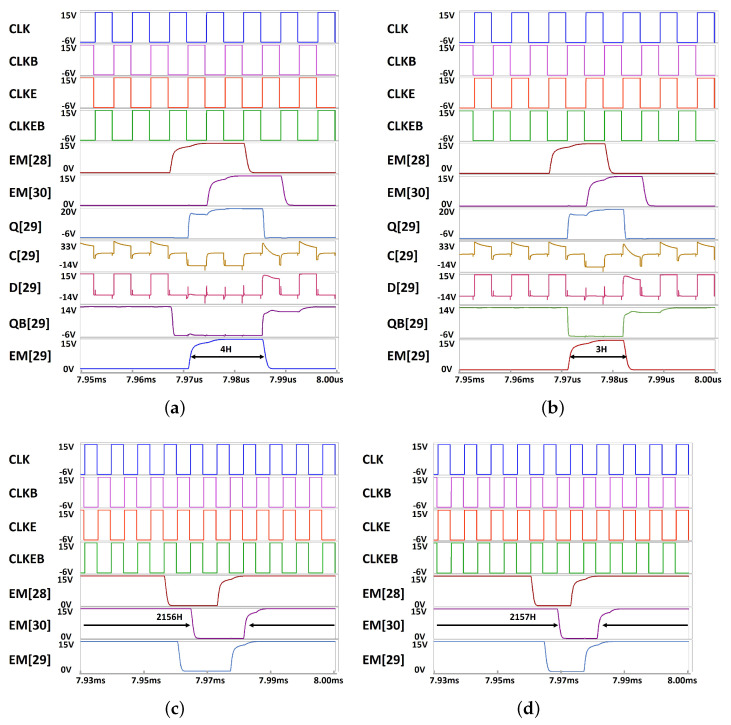
Simulated waveforms of signals, outputs, and internal nodes. (**a**) For the even multiple short pulse width of 4 horizontal times, (**b**) For the odd multiple short pulse width of 3 horizontal times, (**c**) For the even multiple long pulse width of 2156 horizontal times, (**d**) For the odd multiple long pulse width of 2157 horizontal times.

**Figure 9:**
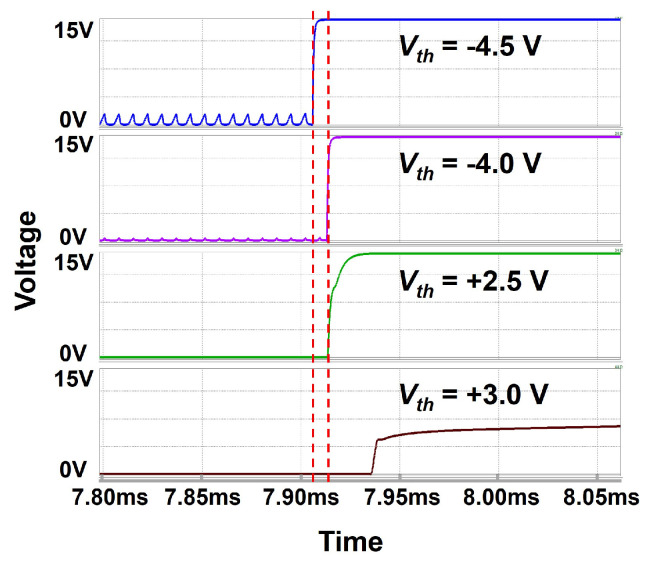
Robustness of the proposed EM circuit over $V_{th}$. The proposed circuit keeps the stable operation over the range of $V_{th}$ from −4.0 V to 2.5 V.

The simulation results are depicted in Figure 8a–d for two short pulse widths of 4 and 3 horizontal times and two long pulse widths of 2156 and 2157 horizontal times in a unit of a horizontal time (H). Each output is shown with the main node (C[n], D[n]) of the improved inverter. The waveforms of the 29-th EM circuit including Q[29], QB[29], C[29], D[29], and EM[29] are verified to accomplish the desired operations for all cases of even and odd multiples as well as short and long pulse widths. In addition, the operation of the proposed circuit is verified to have the robustness over the wide range of $V_{th}$ variance from −4.0 V to 2.5 V. As plotted in Figure 9, while the lower $V_{th}$ than −4.0 V causes the wrong rising transition at EM[n], the higher $V_{th}$ than 2.5 V shows the insufficient driving capability.

**Figure 10:**
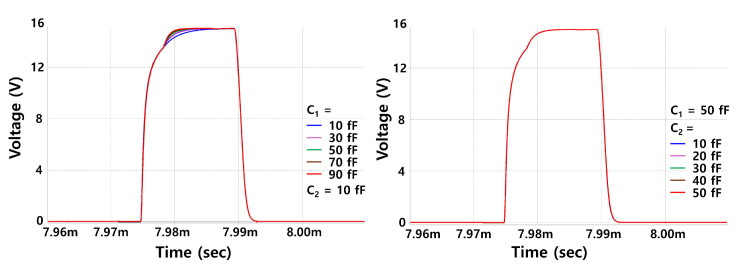
Rising and falling transitions of EM[n] pulses depending on $C_{1}$ and $C_{2}$. Consequently, the transition characteristics are independent of $C_{1}$ and $C_{2}$.

The rising and falling times are estimated as 3.36 μs and 1.57 μs. Those transition characteristics are independent of $C_{1}$ and $C_{2}$ as shown in Figure 10. In particular, the rising transition is conducted with two steps of pre-charging and capacitive coupling and the rising time is determined only by the pre-charging period. C2 contributes only to the holding operation of C[n] and D[n].

**Figure 11:**
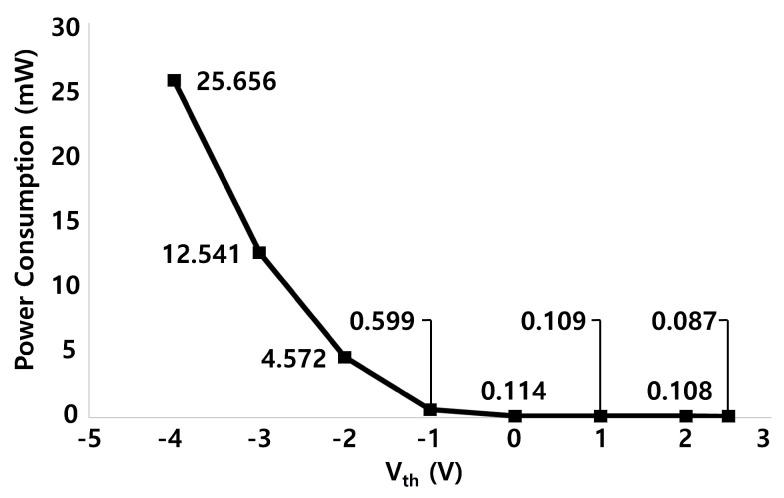
Estimated power consumption over the $V_{th}$ variation from −4.0 V to 2.5 V for 30 stages and the pulse width of 2157 horizontal times.

The power consumption is also evaluated over pulse widths of 3 to 2157 horizontal times as summarized in Table 2 with consideration on the depletion-mode operation of a-IGZO TFTs. The power consumed at VGL1 is always zero because the voltage level is 0 V. The most power is consumed in VGH and VGL2 to generate Q[n], QB[n], and CR[n]. As the pulse width gets longer, the power consumption at VGH and VGL2 becomes smaller. Because STT structures are not employed in two pulling-up TFTs of N3 and N15, the leakage current takes place from VGH to VGL2 during the Pulling-Down period which length increases as the pulse width decreases. The same can be said regarding CLKE and CLKEB since QB[n] nodes are driven by CLKE and CLKEB during the Pulling-Down period. However, the power consumption at CLK and CLKB is larger for longer pulse widths due to their charging operation to A[n] for the high period of EM[n − 1]. As a result, the power consumption is managed at the low values from 0.836 mW to 0.568 mW. In addition, we sift through the power consumption for 30 stages and the pulse width of 2157 horizontal times over the variation of $V_{th}$ from −4.0 V to 2.5 V as presented in Figure 11. As expected, as $V_{th}$ moves in the negative direction, the power consumption goes up due to the increased leakage current.

**Figure 12:**
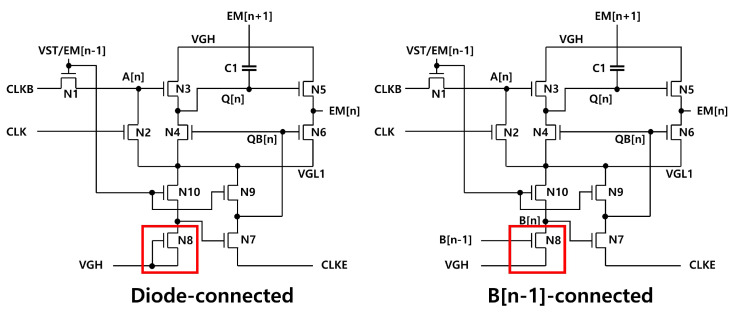
Estimated power consumption over the $V_{th}$ variation from −4.0 V to 2.5 V for 30 stages and the pulse width of 2157 horizontal times.

Lastly, the power consumption is compared with EM pulse circuits of the diode-connected load and the B[n − 1]-connected load inverters of Figure 12 as presented in Table 3, where a-IGZO TFTs at the enhancement-mode ($V_{th}$ = 1.0 V) are employed to exclude the leakage current effects. As a result, it is ensured that the proposed circuit consumes the lower power than two others for most pulse widths. Because QB[n] is disconnected from CLKE and CLKEB during the high EM pulse period, the power consumption of the B[n − 1]-connected circuit is slightly lower than the proposed one at the long pulse width of 2157 horizontal times.

**Figure 13:**
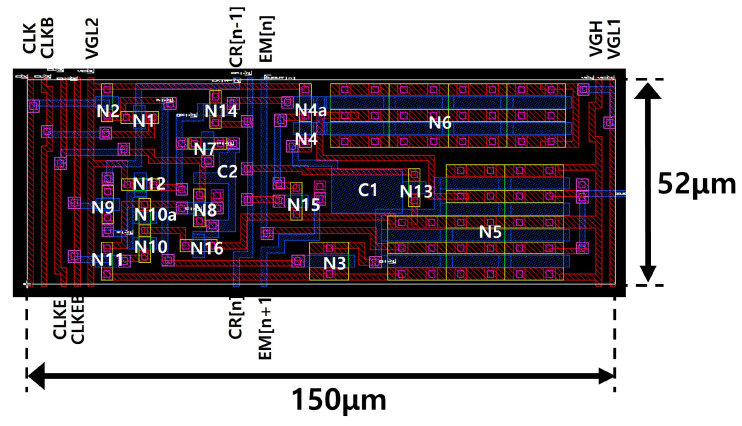
Layout design of the proposed EM pulse generation circuit.

The layout design is illustrated in Figure 13 with the area of 150 μm × 52 μm that corresponds to 488 pixel per inch (PPI) in the vertical direction. When gate driver circuits are implemented in both sides of the panel, this EM circuit can support up to 976 PPI that is higher than 721 PPI of the 6.1-inch UHD panel.

## 4. Conclusions

We demonstrate a low power EM generation circuit that significantly improves the power consumption of an internal inverter for QB[n] generation to control pulling-down TFTs. The proposed inverter effectively eliminates the shoot-through current paths and the leakage current due to the depletion-mode operation of a-IGZO TFTs is addressed by two low supply voltages and STT schemes. The proposed circuit features the robustness over the wide $V_{th}$ variation from −4.0 V to 2.5 V and the low power consumption of 0.836 mW for the short pulse width of 3 horizontal times and 0.568 mW for the long pulse width of 2157 horizontal times. Additionally, it is ensured that, compared to the circuits with diode-connected load and B[n − 1]-connected load inverters, the proposed circuit achieves the lower power consumption that shows no correlation on pulse widths. Because the inverter is one of basic components in the TFT integrated circuits, the proposed approach without any occurrence of shoot-through current paths substantially contributes to the low cost and low power implementation for the future displays.

## Figures and Tables

**Table 1:** Channel widths of TFTs in a proposed EM pulse generation circuit.

TFT	Width	TFT	Width	TFT	Width
N1	3 μm	N2	3 μm	N3	9 μm
N4	3 μm	N4a	3 μm	N5	150 μm
N6	120 μm	N7	3 μm	N8	3 μm
N9	3 μm	N10	3 μm	N10a	3 μm
N11	3 μm	N12	3 μm	N13	3 μm
N14	3 μm	N15	3 μm	N16	3 μm

**Table 2:** Measured power consumption over pulse Widths for the proposed EM circuit at $V_{th}$ of −0.65 V.

Pulse Width (H)	3	100	201	1000	2157
VGH (mW)	0.575	0.562	0.556	0.493	0.399
VGL1 (mW)	0.000	0.000	0.000	0.000	0.000
VGL2 (mW)	0.224	0.219	0.215	0.184	0.137
CLK/CLKB (mW)	0.001	0.002	0.002	0.005	0.009
CLKE/CLKEB (mW)	0.036	0.035	0.035	0.031	0.023
Total (mW)	0.836	0.818	0.808	0.713	0.568

**Table 3:** Power consumption comparison of the EM circuits with diode-connected load, B[n − 1]-connected load, and proposed inverters at $V_{th}$ of 1.0 V.

Pulse Width (H)	3	100	201	1000	2157
Diode-connected (mW)	0.176	0.335	0.501	1.820	3.711
B[n − 1]-connected (mW)	0.175	0.169	0.162	0.110	0.032
Proposed (mW)	0.037	0.037	0.038	0.040	0.043

## Data Availability

Data are contained within the article.
